# Mindful Eating and Active Living: Development and Implementation of a Multidisciplinary Pediatric Weight Management Intervention

**DOI:** 10.3390/nu12051425

**Published:** 2020-05-14

**Authors:** E. Thomaseo Burton, Webb A. Smith

**Affiliations:** 1 Pediatric Obesity Program, Department of Pediatrics, College of Medicine, University of Tennessee Health Science Center, Memphis, TN 38163, USA; eburto10@uthsc.edu; 2 Children’s Foundation Research Institute, Le Bonheur Children’s Hospital, Memphis, TN 38103, USA

**Keywords:** pediatric obesity, mindfulness, cooking class, group intervention, family-based

## Abstract

Pediatric overweight and obesity are significant individual and public health issues that require an innovative approach. While evidence suggests that intensive family-based behavioral lifestyle modification can improve weight status, practical and logistical realities limit the ability of primary healthcare providers to intervene effectively. MEALs (Multidisciplinary Engagement and Learning/Mindful Eating and Active Living) is a family-based mindfulness intervention developed to address pediatric overweight and obesity, while improving healthy lifestyle behaviors through cooking classes. The incorporation of mindfulness, a psychological strategy associated with increased awareness of internal experiences, allows for a focus on the importance of healthy eating along with safe and efficacious kitchen practices. The Template for Intervention Description and Replication (TIDieR) checklist and guide is used to describe the intervention with the intention of providing necessary details to implement the intervention in clinical practice or replicate the intervention for further study. Lessons learned from pilot iterations of the intervention are provided.

## 1. Introduction

Overweight and obesity are among the most pressing health issues facing children and adolescents today. In fact, nearly one third of youth in the United States have overweight or obesity, which increases their lifelong risk for chronic physical (e.g., type 2 diabetes, hypertension), emotional (e.g., depression, anxiety) and social (e.g., teasing/bullying, discrimination) health concerns [1,2,3]. The individual and public health burden of pediatric overweight and obesity signal a need for development, implementation, and evaluation of efficacious prevention and intervention strategies [4].

The United States Preventive Services Task Force (USPSTF) recommends that the treatment of pediatric obesity begin with intensive behavioral intervention, before pharmacological and surgical interventions are considered [5]. In their recommendation statement, the USPSTF reports that interdisciplinary family-based pediatric weight management can lead to improvements in weight status and cardiometabolic outcomes among children and adolescents with obesity, particularly in higher-intensity (≥26 total contact hours) interventions. However, the mounting evidence supporting the efficacy of behavioral interventions is confounded by a lack of data on how these interventions function in high-risk and under-resourced populations [6]. Furthermore, pediatric weight management programs struggle with attrition [7], highlighting the importance of patient engagement in prevention and intervention efforts.

While dietary, physical activity, and sedentary behaviors are important contributors, overweight and obesity are far more complex than individual lifestyle choices [8]. Historical, economic, social, and cultural factors are also implicated, and must be acknowledged in order to gain a comprehensive understanding of these health conditions. Despite recent evidence of stabilization of this epidemic, rates of overweight and obesity remain disproportionately high among certain racial and ethnic minority youth, namely Hispanics and non-Hispanic Blacks [9,10,11]. Furthermore, the severity of obesity tends to be greater among youth of color [4]. Socioeconomic status is also a strong predictor of obesity prevalence [12,13]. Taken together, these disparities indicate an urgent need for the development of culturally responsive interventions for child and adolescent overweight and obesity [14,15].

Food preparation and eating preferences bear strong cultural underpinnings and are thus an ideal focus for behavioral weight management intervention [16]. Cooking classes have become a popular way to promote healthy eating and empower individuals and families to incorporate healthy behaviors. However, little research has been conducted on the interventional elements of cooking classes that effect positive change [17]. Furthermore, few cooking classes have taken a family-based approach [18].

Because culture is so engrained in behavior, it is critical that overweight and obesity management interventions consider community environment, practices, and beliefs. The present manuscript describes the development and implementation of a culturally tailored group-based intervention to manage excess weight among children and their families. Considering the links between physiological stress and weight [19], mindfulness is a potential mediator of weight change that fits well within the framework of behavioral weight management. Mindfulness is not simply a relaxation technique. Rather, it is a psychological strategy that focuses on directed attention and awareness of internal experiences (e.g., emotions, physical sensations) [20,21,22] and has been associated with cardiometabolic improvements in adults as well as youth [23,24,25]. Specifically, mindfulness involves paying attention intentionally, presently, and non-judgmentally. The practice of mindfulness skills, such as meditation, breath awareness, and mindful eating have been shown to facilitate greater awareness to feelings of hunger and cravings for high sugar/high fat foods, as well as more adaptive coping with psychological distress that may prompt emotional eating patterns [26,27]. In terms of cooking, mindfulness has numerous applications, from grocery shopping (attention to costs and nutritional quality of food being purchased) to food preparation (awareness of kitchen safety) and consumption (attention to serving sizes and satiety cues).

The purpose of this manuscript is to describe the development and initial implementation of MEALs (Multidisciplinary Engagement and Learning/Mindful Eating and Active Living), a family-based mindfulness intervention developed to address pediatric overweight and obesity while improving healthy lifestyle habits via cooking classes. Behavioral interventions are often not developed or implemented in a systematic fashion. Furthermore, interventions reported in peer-reviewed manuscripts tend to focus on statistical results, which leaves little space to provide details of the intervention itself [28]. This practice does not allow for reliable implementation into clinical practice or replication for research [29,30]. The refining of interventions through replication is necessary to determine the most efficacious elements, and hence there is a move towards reporting intervention details in a separate article [31]. As such, the aim of the present article is not to present statistical data. Rather, the ‘data’ are details of the intervention.

A number of public health frameworks have been proposed to address the need for a more nuanced description of the intervention development, implementation, and evaluation process. Among these are intervention mapping [32], the RE-AIM (Reach, Effectiveness, Adoption, Implementation, Maintenance) Framework [33], logic models [34], and the Medical Research Council (MRC) Framework [35]. This current description of MEALs is guided by the Template for Intervention Description and Replication (TIDieR) [29] checklist and guide, an extension of the Consolidated Standards of Reporting Trials (CONSORT) [30] and Standard Protocol Items (SPIRIT) [36] statements. Specifically, TIDieR was developed to guide and improve the quality of reporting on interventions by ensuring transparency [28]. The checklist itself includes 12 items that formally delineate specific elements of an intervention.

## 2. Description of MEALs Using the TIDieR Checklist

The Administrative Section of the University of Tennessee Health Science Center (UTHSC) Institutional Review Board (IRB) approved the study procedures described below (45 CFR 46.404). Written consent and assent were obtained from parents or legal guardians and child participants, respectively. 

### 2.1. BRIEF NAME: Provide the Name or a Phrase that Describes the Intervention

The title of the present intervention is MEALs, which is an acronym for Mindful Eating and Active Living/Multidisciplinary Engagement and Learning. This name acknowledges the holistic approach required to address pediatric overweight and obesity. Although MEALs is a series of cooking classes, the intervention offers multiple opportunities to examine the role of mindfulness, from awareness of hunger and satiety cues to quality and quantity of food, and the importance of safety in the kitchen.

### 2.2. WHY: Describe Any Rationale, Theory, or Goal of the Elements Essential to the Intervention

MEALs was developed in response to feedback provided by patients and families enrolled in the Healthy Lifestyle Clinic, a pediatric weight management clinic at Le Bonheur Children’s Hospital in Memphis, TN [37]. In a series of focus groups, caregivers expressed a desire for more experiential components in the management of their children’s weight and related comorbidities. One caregiver stated, “I like the nutrition part…maybe a cooking class for the kids…I notice that when she helps cook the food, she’ll eat it better…because she has a responsibility for it.” Another caregiver stated, “well, lately I been cooking, trying to do this budget thing, but it’s kind of easier to do a home cooked meal where you can have left over (food) for tomorrow. I just need help to know what’s good to cook and how to cook it.” Evaluation of these expressed needs led the PIs to develop an intervention that was relevant and responsive to the Healthy Lifestyle Clinic population, which is largely African American and from under-resourced communities.

The social-ecological model [38,39] illustrates the multifactorial influences on a child’s weight status and highlights that behavioral interventions must consider the interplay of individual, familial, community, and societal contexts that inform lifestyle practices and beliefs. In pediatric populations, patient and family prioritization of health, financial, and cultural needs underscore the importance of developing engaging, meaningful, and relevant content [40], and are prime targets for mindful awareness. Furthermore, experiential (i.e., hands-on) education around lifestyle practices has been found to be more engaging for patients and families when compared to traditional counseling [41,42]. Finally, in light of rising healthcare costs associated with overweight and obesity, group-based interventions may be a cost-effective means of delivering intensive interdisciplinary treatment [43]. Food preparation and eating preferences bear strong cultural underpinnings, and thus they are ideal foci of lifestyle intervention.

MEALs was developed to address common limitations in traditional management of pediatric overweight and obesity. Families are typically instructed to make changes to diet and exercise [37], though they may not have the skills or resources to incorporate these changes. Social cognitive theory [44], which describes the interaction of individual, environmental, and behavioral influences on health outcomes, provided additional guidance for the development of the current intervention. This theoretical framework has been used to predict dietary behavior change in children and adults [45,46], and evaluates mechanisms of behavior change, such as self-efficacy (an individual’s confidence in their abilities), behavioral capability (an individual’s actual ability to apply knowledge and skills to engage in a behavior), and observational learning (an individual’s ability to reproduce a behavior after having seen it demonstrated).

Taken together with other social cognitive constructs (i.e., social support, goal-setting) that have been used to develop successful behavioral interventions, MEALs seeks to blend educational and experiential learning by implementing a mindfulness- and family-based intervention to address excess weight, while improving healthy lifestyle habits via cooking classes. Specifically, anticipated outcomes and impacts of the intervention include improved knowledge and attitudes related to healthy behaviors in the short-term and mindful incorporation of these healthy behaviors in the mid-term. Long-term anticipated outcomes include changes in body composition and overall improvements in cardiometabolic health.

### 2.3. WHAT: Describe Materials and Procedures 

MEALs is a family-based cooking and education program that combines group-based instruction and experiential learning to address attitudes and behaviors as they relate to healthy lifestyle choices. The program consists of one two-hour cooking and education session per week for three weeks, each lasting approximately 120 min (see Table 1). Each session follows the same format: education (20 min); taste test (10 min); hands-on meal preparation (30–40 min); an opportunity to ‘dine and discuss’ (30–40 min); and concluding comments and clean-up (10 min). Participants are a caregiver-child dyad.

As can be seen in Table 1, the education portion of each session includes a didactic lecture covering a healthy lifestyle topic in addition to a kitchen safety skill. Healthy lifestyle topics include nutrition, physical activity, electronic screen time, and sleep guidelines, while kitchen safety skills cover the proper handling of knives, hand washing, and cross contamination. Mindfulness, which is the continuous thread through the sessions, is imparted in both a didactic and applied fashion. For example, Session 1 introduces the concept of mindfulness (i.e., being aware of thoughts, feelings, and physical sensations in the present moment, without judgment and without trying to change) and links it to hunger and satiety cues (i.e., mindful eating), as well as safe chopping and cutting techniques.

The taste test is another application of mindfulness skills. In this exercise, participants are exposed to a variety of sample-size food items (prepared in real-time by the group co-facilitator) and encouraged to share feedback on the appearance, smell, texture, and taste of these foods. This includes prompts to engage with the items beyond initial like or dislike. Participants are also encouraged to make suggestions of how the item they sampled could be improved in a way that is consistent with a healthy lifestyle. It is important to note that each taste test item was mindfully selected in consideration of costs, safety concerns, varying access to cooking equipment, and likelihood of families incorporating these items into their dietary routine. As such, each taste test includes a no cook recipe, a microwave recipe, and a recipe that is prepared on the stovetop or in the oven (see Table 1). 

Following the taste test, participants wash their hands, don an apron, and move to their work stations for hands-on meal preparation. Stations are positioned around a standing height island/table top, with all stations facing the stove and facilitator. A caregiver and youth are positioned at the same station and work together to complete the recipes. 

**Figure 1:**
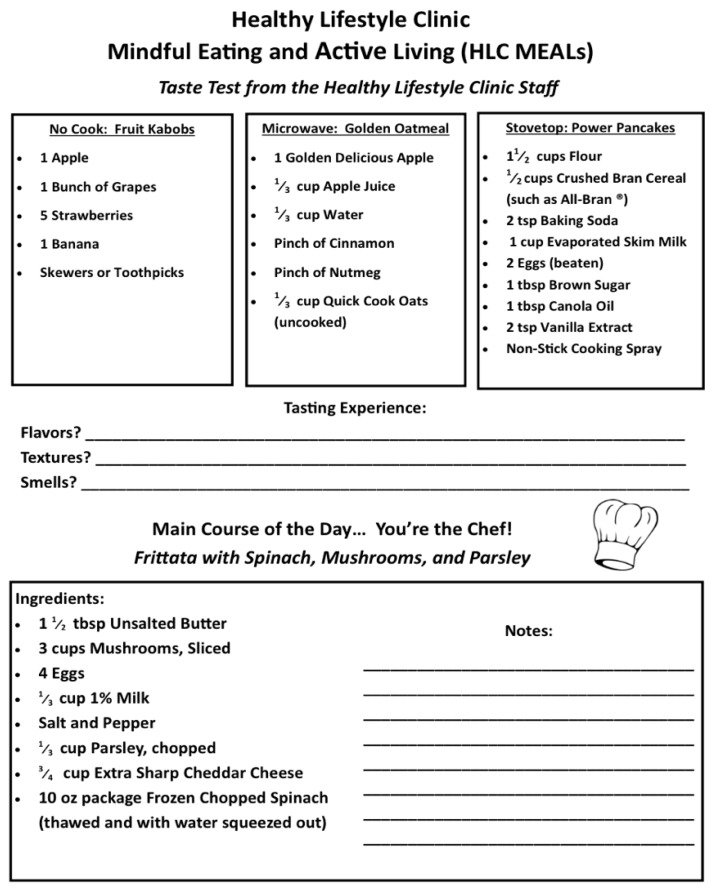
Sample menu card distributed to meals (multidisciplinary engagement and learning/mindful eating and active living) participants.

**Figure 2:**
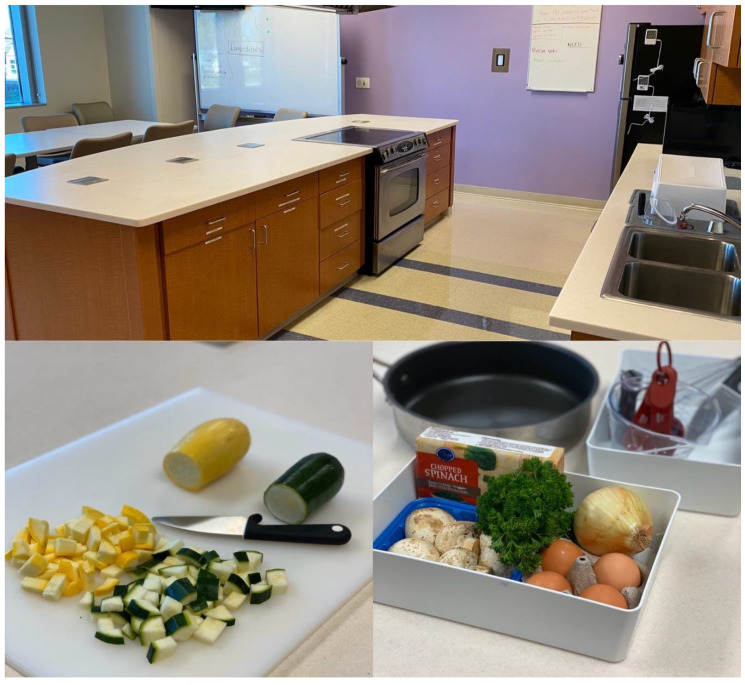
View of cooking area (top center); prepared ingredients and cooking utensils (bottom left and right).

Laminated menu cards, with descriptions on one side and recipes on the other, are placed at each station (see Figure 1). Each dyad has approximately three feet of countertop space, which is adequate for all food preparation and cooking, yet close enough that families interact and a facilitator can easily see and supervise all participants. All ingredients necessary to prepare the meal are pre-portioned and placed in 10-quart plastic tubs at each station, along with a second 6-quart tub with all needed utensils (see Figure 2). The tubs are important for space management and organization, as well as a place to store any trash and dirty utensils for easy clean up. 

Facilitators rotate their focus from dyad to dyad, providing support for families needing more assistance and encouragement to those who are operating more autonomously. As food preparation is completed and ready for the cooking stage, facilitators also operate and manage the stovetop/oven for safety and to ensure that all participants are progressing at a similar pace. While the cooking is finishing, participants are guided to clean their workspace and prepare the place settings for the plating and serving of the food. When cooking is complete, participants are encouraged to plate the food carefully, being mindful that each recipe serves four. Participants are also encouraged to note mindfully other qualities of the food, such as appearance and smell.

The final programmatic portion of MEALs is Dine and Discuss. When sitting down to eat, participants are seated in a way that facilitates active discussion and ensures that no one’s back is to another table. Each table is arranged with four full place settings consisting of a placemat, glassware (plates, bowls, tumblers), silverware, napkin, and water pitchers, and the setting is intentionally formal to create the feel of a special dining experience. Before eating the food that they have just prepared, participants are reminded to take a mindful first bite. The facilitators then prompt discussion about the food, its preparation, and how to translate the knowledge and skills acquired in the session to the participants’ home life. In particular, participants are guided to provide thoughtful and specific feedback on what they liked and did not like about the food. Facilitators are trained to counter non-specific responses like, “I don’t like it”, with probing questions to elicit more in-depth commentary on specific critiques about appearance, taste, texture and smell and how these elements can be improved.

Other discussion prompts include the economics of healthy food shopping and preparation (particularly affordability), comparison of the MEALs menu to more traditionally preferred food choices, and the practicality of incorporating menu items at home. At the conclusion of each session, a brief summary is provided, along with encouragement to practice and apply mindfulness skills in everyday life. Participants receive copies of all recipes, including the taste test samples, and leftover portions are boxed for participants to take home. Finally, participants straighten their areas, discard trash, and return service items for cleaning.

The facilitators then prompt discussion about the food, its preparation, and how to translate the knowledge and skills acquired in the session to the participants’ home life. In particular, participants are guided to provide thoughtful and specific feedback on what they liked and did not like about the food. Facilitators are trained to counter non-specific responses like, “I don’t like it”, with probing questions to elicit more in-depth commentary on specific critiques about appearance, taste, texture and smell and how these elements can be improved.

Other discussion prompts include the economics of healthy food shopping and preparation (particularly affordability), comparison of the MEALs menu to more traditionally preferred food choices, and the practicality of incorporating menu items at home. At the conclusion of each session, a brief summary is provided along with encouragement to practice and apply mindfulness skills in everyday life. Participants receive copies of all recipes, including the taste test samples, and leftover portions are boxed for participants to take home. Finally, participants straighten their areas, discard trash, and return service items for cleaning.

In order to assess intervention-associated outcomes, a number of measures are administered to MEALs participants pre- and post-intervention. As can be seen in Table 2, caregiver and child anthropometrics, body composition, and vital signs are measured at baseline and 0, 3, and 6 months after completion of the intervention. Participants also complete questionnaires assessing mindfulness, quality of life, and stress and hassles. In terms of behavior change, social cognitive constructs (e.g., self-efficacy, behavioral capability) are measured through assessment of knowledge, attitudes, and behaviors related to health and lifestyle choices at these same timepoints. Finally, questions designed to assess feasibility and acceptability are administered at the final post-intervention follow-up. 

### 2.4. WHO PROVIDED: Describe the Expertise, Background, and Specific Training Given to Each Category of Intervention Provider

Each intervention session is led by facilitator and co-facilitator. While the facilitator leads the mindfulness and didactic activities and demonstrates techniques related to food preparation, the co-facilitator provides the more supportive role of preparing taste test recipes and managing flow in the kitchen (e.g., organizing food preparation areas). Co-facilitators also model participation when families are hesitant to engage. Both facilitators and co-facilitators interact with the families around cooking to encourage engagement, monitor for safety concerns, and foster a robust discussion.

In pilot trials of MEALs, the facilitator was a trained research assistant and co-facilitators were advanced graduate students in psychology and dietetics. Training was conducted by study PIs, who provided education on nutritional guidelines, food preparations, and the principals of mindfulness. It is important to note that study PIs included a licensed psychologist with formal training in mindfulness based stress reduction [55] and dialectical behavior therapy [56], of which mindfulness is a core component. Facilitator training was girded by an intervention manual, which provided detailed instructions for each component of the intervention. 

### 2.5. HOW: Describe the Modes of Delivery of the Intervention

The intervention is designed to be delivered in face-to-face group sessions. Initial iterations ranged in size from three to five caregiver-child dyads per session.

### 2.6. WHERE: Describe the Type(s) of Location(s) Where the Intervention Occurred, Including Any Necessary Infrastructure or Relevant Features

MEALs can be delivered in any private space with access to chairs, tables, audiovisual equipment and kitchen equipment (i.e., stovetop, refrigerator, cookware). The intervention was developed and initially delivered at an urban pediatric healthcare center that serves children and families from culturally diverse backgrounds. Initial iterations of the intervention were held in a hospital-based demonstration kitchen.

### 2.7. WHEN and HOW MUCH: Describe the Number of Times the Intervention Was Delivered and over What Period of Time

Each iteration of MEALs is one two-hour session per week for three consecutive weeks, with each session lasting approximately two hours. MEALs sessions are offered on a variety of days and times to maximize participant accessibility. Sessions were offered during evening hours (i.e., 5:00 pm to 7:00 pm) to coincide with a typical dinner time. Further, sessions were scheduled on different nights of the week to minimize conflict with other standing activities, such as church, youth sports, and school obligations. To date, MEALs has enrolled 10 cohorts, with each cohort comprising approximately four caregiver-child dyads.

### 2.8. TAILORING: If the Intervention Was Planned to Be Personalized, Titrated or Adapted, then Describe What, Why, When, and How

In its initial conception, MEALs was tailored to appeal to a specific demographic, namely African Americans living in under-resourced communities in the southern region of the United States. This cultural tailoring was evidenced mainly in recipe selection; recipes can certainly be adapted to suit participants from a variety of backgrounds. In terms of more individualized tailoring, food allergies and dietary restrictions were assessed during the intake, and appropriate changes were made to planned menu items. For example, participants who reported lactose intolerance were provided dairy-free alternatives to use in their recipes.

### 2.9. MODIFICATIONS: If the Intervention Was Modified during the Course of the Study, Describe the Changes (What, Why, When, and How)

No modifications were made during pilot trials. However, lessons learned during initial implementation are outlined in the discussion.

### 2.10. HOW WELL: Planned and Actual Fidelity to the Intervention

MEALs is a manualized intervention, with specific didactic and experiential components for each session. Facilitators and completed checklists at the conclusion of each of each session to assess fidelity to the manual.

## 3. Discussion

Effective treatment of pediatric overweight and obesity requires intensive intervention to address modifiable lifestyle behaviors [5]. Furthermore, the research suggests that involving the family in weight management may be especially beneficial for racial and ethnic minority youth, who are at greater risk [57]. However, limited time, resources, and expertise have impeded traditional efforts at managing excess weight in youth, and many weight management interventions fail to consider culturally relevant elements that may make intervention more engaging for youth and their families [58,59]. The MEALs program is an interdisciplinary intervention developed to deliver health education on pediatric overweight and obesity, while improving healthy lifestyle habits via hands-on cooking classes. Guided by the TIDieR checklist, this manuscript aims to provide sufficient detail to allow clinicians and researchers to replicate the intervention for additional study.

To our knowledge, MEALs is the first mindfulness-based cooking class developed to address overweight and obesity in youth. Rather than focusing solely on dietary practices, the intervention includes a more holistic, multidisciplinary approach to healthy lifestyle changes. In particular, the overlay of mindfulness skills acknowledges the role of behavior in weight management, while making a potentially abstract concept more accessible for youth and their families. Future studies should assess participant perceptions of the cultural relevance of the intervention.

### 3.1. Modifications and Suggestions Based on Lessons Learned

In its current form, MEALs is not yet empirically validated, which underscores the importance of fully describing the intervention for further study in larger and more robust trials. However, anecdotal feedback garnered during pilot trials suggests that the intervention has strong promise for further dissemination. In addition to positive comments and requests from participants for extended content and additional sessions, participation was high and attrition was low. Nevertheless, reflection on these pilot trials uncovered several programmatic modifications that may enhance the overall quality of MEALs in future iterations.

#### 3.1.1. Costs and Resources

First, we learned that participants have varying and inconsistent access to food, both in terms of costs and availability. Considering the demographics of populations most affected by pediatric obesity [4,37], we did anticipate that many families have limited budgets to feed their families, and addressed this by implementing a cost-per-serving limit when developing recipes. We also incorporated strategies to maximize budgets by using frozen and canned items, purchasing store brand foods, attending to store sales and specials, and when possible, buying bulk quantities and storing for later by freezing or partitioning. Despite these a priori efforts, many caregivers still expressed concerns about not only the costs of recipe items, but also barriers to actually obtaining items (e.g., lack of reliable transportation, no grocery stores within proximity to home). These responses led us to refine facilitator discussion points of barriers, such as cost and ways to utilize the skills presented as a robust group discussion, where families were encouraged to offer their own practical solutions to participants. 

In a related vein, we also confirmed that many families live with limited cooking capacity (e.g., no working stove or oven). More commonly, participants reported that children are responsible for their own meals, due to caregivers working late shifts and/or multiple jobs. While we preemptively included no cook and microwave recipes in each session, more prompted discussion on addressing and overcoming some of these access issues will be important in future iterations of MEALs. It is important to note, however, that many of these issues arose during the Dine and Discuss segment, which facilitated peer-level discussions around problem solving.

#### 3.1.2. Balanced Approachability of Recipes

Planning and preparing healthy foods can be a daunting task, particularly for those lacking experience and confidence. Many participants expressed concern that cooking at home is challenging and indicated that balancing costs, time, and healthfulness with family food preferences is intimidating. An overarching intention of MEALs is to present healthy cooking as an approachable option for improving health. In terms of recipes, we sought to challenge participants with healthier options, while still presenting dishes that were familiar to the majority of participants. We attempted to address the intimidation factor by introducing basic kitchen skills that have multiple applications (e.g., proper knife handling), demonstrating cooking techniques, and providing detailed instructions and assistance for the experiential portion. While this approach did seem to engender confidence in many participants, we realized that some families will need more support than others. For those participants requiring more individualized assistance, the co-facilitator was able to spend more time introducing the systematic nature of recipes and time management with gentle prompts to check time and progress, as well as provide more explanation and demonstration.

#### 3.1.3. Engagement of the Dyad

A family-based intervention must be applicable and engaging, across multiple developmental stages and life experiences. Although caregivers tend to be the primary decision makers regarding food shopping and food preparation, we contend that children and adolescents can play a more active role in household-level healthy decision making. Moreover, youth will become increasingly more responsible for their health over time and will one day have to manage households of their own. As such, MEALs seeks to engage both caregiver and child in meaningful and appropriate ways. For example, youth are tasked with setting the table and arranging and measuring ingredients, while caregivers are responsible for cutting, chopping, and handling hot cookware. Highlighting the valuable and necessary contributions of both members fostered a teamwork mentality and allows dyads to depend on each other to prepare and serve the meal.

We did learn that some caregivers find it difficult to relinquish their traditional responsibilities, while others need encouragement to be more involved in the process. In fact, we noticed that many participants were reluctant to detach from their electronic devices (i.e., cellular phones) for the 2-h duration of the session. In response, we put very simple rules encouraging the limited use of electronics during sessions and developed defined roles that were applied to encourage equal participation of key tasks. Over the course of the intervention, these were progressed to more formally emphasize phone-free engagement in the food preparation process, as another example of mindfulness. 

#### 3.1.4. Group Support and Discussion 

As a group-based intervention, each iteration of MEALs brings together multiple caregiver and child dyads to learn, work, and eat together. We anticipated that the dynamics of a group would lend to exchange of ideas and hoped that each cohort would find some common bonds. What we learned is that the group-based format also created space for discussion about how families can adapt the skills learned to their real-world scenarios and experiences. Participants commiserated around the challenges of managing multiple tasks and barriers, such as cost and busy schedules, and engaged in robust conversations about the appealing and unappealing aspects of recipes, and in particular, ideas on how to modify recipes based on their likes and dislikes. 

As with any group, some participants are more outgoing than others. Facilitators worked to engage all participants in group discussions, while not making anyone feel pressured to participate or singled out as a negative example. This included prompts for youth to interact with the larger group as well. While we wanted youth participants to hear and understand caregiver perspectives, we also wanted caregivers to be mindful of their children’s viewpoints. 

#### 3.1.5. Personnel Concerns

Facilitators and co-facilitators are the face of MEALs. One of the most meaningful lessons learned in the pilot iterations of MEALs had to do with staffing. We quickly learned that a base level of skills and comfort are necessary to run a productive session. Foremost, although all MEALs recipes were developed with approachability in mind, the ability to demonstrate and coach these recipes is contingent on basic cooking skills. The intervention also requires staff to multitask, shift attention, and respond to the needs of participants. As a practical solution, we developed roles for the intervention teams that paired a staff member with a high level of comfort with multitasking and facilitating with a staff member more comfortable performing a specific task-based instruction. 

In addition to the hands-on aspects of facilitation, staff must manage the flow and pace of tasks and discussions. For example, considering the importance of keeping the session running on time, discussions should be topically consistent and relevant to the goals of the program. We learned that facilitators and co-facilitators play an important role in addressing and interjecting important information, such as when adjustments to recipes would be suitable and when a healthier choice could be made. Additionally, staff must feel empowered to redirect inappropriate or off-task commentary. Finally, because MEALs is a mindfulness-based intervention, some familiarity with and appreciation for mindfulness is important. To ensure staff understood how to intentionally facilitate the mindfulness components of the intervention, personnel were introduced to mindfulness concepts and completed training with an experienced psychologist. While personnel do not need to be experts in mindfulness, a key role for staff is fostering the development and application of these skills across all aspects of the intervention.

## 4. Conclusions

The increasing prevalence and severity of pediatric overweight and obesity signal the need for innovative approaches to address the short- and long-term costs associated with the disease process. Because interdisciplinary care of pediatric obesity tends to exist in limited contexts, it is important to extend efficacious interventions to community-based providers. It is critical that such interventions be thoroughly vetted for accessibility, sustainability, and equitability. The TIDieR Checklist offers a useful framework for describing the details of MEALs which will allow for replication and further refinement of the intervention.

## Figures and Tables

**Table 1:** Summary of MEALs educational objectives and recipes.

	Education	Exposure		Experiential
Session 1: Breakfast	Topic: Behavioral Health Introduction to Mindfulness Hunger and Cravings: Paying Attention to the Difference Food Handling Safety Knife Safety	No Cook	**Fruit Kabobs** fresh fruit arranged on a skewer	**Breakfast Frittata** Eggs baked with spinach and mushrooms, garnished with cheese.
		Microwave	**Golden Oatmeal** apples, cinnamon, and oats served warm	
		Stove/Oven	**Power Pancakes** fluffy griddle cakes made from bran and whole wheat flour	
Session 2: Lunch	Topic: Nutrition Review of Mindfulness Recognizing foods that are better choices for health: Go, Slow, and Whoa! Reading nutrition labels Review of food and kitchen safety	No Cook	**Peach Banana Freeze** blended smoothie of frozen fruit, spinach, and almond milk	**Roasted Vegetable Pizza** Oven-roasted yellow and zucchini squash on whole wheat flatbread. Served with green salad and participant-prepared vinaigrette.
		Microwave	**Sweet Potato Chips** thinly sliced yams crisped in the microwave	
		Stove/Oven	**Roasted Almonds** warmed nuts tossed with rosemary	
Session 3: Dinner	Topic: Exercise Review of Mindfulness Having Fun While Being Active: Movement is Medicine Body positioning and space in the kitchen Review of food and kitchen safety	No Cook	**Hummus and Crudités** prepared hummus accompanied by carrot and celery sticks	**Tuna Cakes** Pan seared water-packed tuna patties accompanied by low-sodium French- style green beans from a can and frozen sweet potato fries. For dessert, Greek yogurt-based cheesecake square with berries.
		Microwave	**Beans and Rice** black beans mixed with brown rice and tomato salsa	
		Stove/Oven	**Chicken Taco** spiced and grilled chicken breast served on a corn tortilla	

**Table 2:** Summary of pre- and post-intervention measurements.

Measurements		Description	Baseline	MEALs Sessions			Post Session Follow up		
				1	2	3	0 Months	3 Months	6 Months
Participants	Informed Consent		X						
	Anthropometrics	Height and weight	X				X	X	X
	Body Composition	Bioelectrical impedance (InBody 770)	X				X	X	X
	Vital Signs	Heart rate and blood pressure	X				X	X	X
Caregiver Surveys	Family Demographic Information		X				X	X	X
	Mindfulness Attention Awareness Scale (MAAS) [47]	15-item survey on day to day experiences and awareness	X				X	X	X
	Attitudes, Knowledge, Behaviors [48,49]	22 questions about health and lifestyle choices	X				X	X	X
	SF-36v2 Health Survey [50]	11- item health-related quality of life survey	X				X	X	X
	Pediatric Quality of Life Inventory, Parent Report [51]	23-item caregiver report on child’s health-related quality of life	X				X	X	X
	Perceived Stress Scale [52]	10-item appraisal of life situations as stressful	X				X	X	X
Youth Surveys	MAAS-Child or Adolescent [22,53]	15 question survey on day to day experiences and awareness.	X				X	X	X
	Attitudes, Knowledge Behaviors [48,49]	22 questions about health and lifestyle choices	X				X	X	X
	Pediatric Quality of Life Inventory, Self Report [51]	11 question health related quality of life survey	X				X	X	X
	Hassles Scale for Children [54]	49-item measure of children’s daily stress	X				X	X	X
	Post intervention questions	8 questions about feasibility, and quality improvement							X
